# Vulnerability factors of problematic cannabis use

**DOI:** 10.1192/j.eurpsy.2025.932

**Published:** 2025-08-26

**Authors:** A. Mhalla, A. Ben Haouala, M. Ben Mbarek, N. Faouel, B. Amamou, F. Zaafrane

**Affiliations:** 1 Laboratory Research LR05E10; 2 Laboratory research LR05ES10, University of Monastir, Monastir, Tunisia

## Abstract

**Introduction:**

The cannabis use has increased widely in Tunisia during the ten past years, especially in adolescents and young adults. Cannabis use can lead to mental and physical health problems.

**Objectives:**

The aim of the study was to determine the factors associated to problematic cannabis use.

**Methods:**

This was a cross-sectional descriptive and analytical study including 115 subjects arrested for suspected drug use, carried out using a pre-established questionnaire, the CAST (Cannabis Abuse Screening Test) for the assessment of cannabis use, the Hamilton Anxiety and Depression scale, the 5-word Test of Dubois for memory evaluation, and the Rosenberg scale for the assessment of self-esteem.

A urine dosage of cannabis was also performed.

**Results:**

The mean age of the study population was 25.19 ± 6.7 years. The sex ratio was 8.16. the problematic users represented 73.9% of the study population. The cannabis was used in the form of joints. Memory decline was noted in 73% of the cases, and 52% of users had anxiety and depression symptoms.

Problematic use was associated to male gender (p<0.001), to an age older than 21.5 years (p= 0.01), to early initiation (p= 0.005), to memory decline (p= 0.003) and the higher scores of anxiety (p< 0.001)

**Conclusions:**

The frequency of cannabis consumption and the vulnerability factors of problematic use must be taken into account in prevention campaigns.

**Disclosure of Interest:**

None Declared

